# The correlates of chronic disease-related health literacy and its components among men: a systematic review

**DOI:** 10.1186/s12889-015-1900-5

**Published:** 2015-06-26

**Authors:** Jeff Davey, Carol A. Holden, Ben J. Smith

**Affiliations:** School of Public Health and Preventive Medicine, Monash University, Lev 6, the Alfred Centre, 99 Commercial Road, Melbourne, VIC 3004 Australia; Andrology Australia, School of Public Health and Preventive Medicine, Monash University, Lev 1, 549 St Kilda Road, Melbourne, VIC 3004 Australia

**Keywords:** Men’s health, Health literacy, Chronic disease, Health behaviours

## Abstract

**Background:**

Chronic diseases drive the burden of disease in many societies, particularly among men. Lifestyle behaviours are strongly associated with chronic disease development, and in a number of countries men tend to engage in more risky behaviours, and have lower health knowledge and attention to prevention, than women. This study investigated the correlates of men’s health literacy and its components about major lifestyle-related diseases, namely ischaemic heart disease and type 2 diabetes mellitus, to gain evidence to guide the development of policy and programs to improve men’s health.

**Methods:**

A systematic review was undertaken of observational studies that investigated men’s health literacy and its components related to ischaemic heart disease or type 2 diabetes mellitus, and their associated risk factors. The Cumulative Index to Nursing and Allied Health Literature (CINAHL), MEDLINE, PsycINFO, Embase and the Cochrane Library databases were searched for articles published since 2003. The strength of the evidence was rated using the GRADE approach.

**Results:**

After screening and review of 504 articles, the search elicited nine studies for inclusion: only one study examined health literacy (nutrition literacy). The majority of included studies focused on only one component of health literacy, namely knowledge (*n* = 7) and personal skills (confidence) (*n* = 1). Twenty correlates were identified, primarily relating to the knowledge component, with the strength of the evidence for only one correlate, education, graded as being of moderate quality. The evidence for all other correlates was graded as being of low quality.

**Conclusions:**

The limited body of research identified may have resulted from a lack of consensus about the definition of health literacy, and a concordant set of validated health literacy measures. Despite these limitations, broadening the search to include components of health literacy has identified that several factors are associated with men’s knowledge and awareness of ischaemic heart disease and type 2 diabetes mellitus that will assist in the development of men’s health promotion strategies. However, addressing the broader knowledge gaps and controversy in the health literacy field will deliver policy and program benefits to address these major contributors to the burden of disease among men.

## Background

It is recognised that in many societies men experience a higher burden of chronic disease (such as lung cancer, ischaemic heart disease (IHD), cerebrovascular disease, type 2 diabetes mellitus (T2DM) and depression) [1] and are more likely to engage in risky lifestyle behaviours (such as tobacco smoking, physical inactivity, risky alcohol consumption and poor diet) [2] than women. Such disparities have led the World Health Organisation and European Commission to call for action to improve men’s health status [3], and for many nations to develop men’s health policies and strategic plans [4, 5].

A range of socio-economic, biomedical and behavioural determinants influence men’s health outcomes and provide opportunities for health promotion action. While the influence of biomedical factors and risky lifestyle behaviours is well established, more recently, health literacy has assumed an increased importance [6]. Health literacy has been identified as a key determinant of health due to its link with behavioural choices and service usage [7]. The relationship between health literacy and behaviours such as tobacco smoking, physical inactivity, risky alcohol consumption and poor diet, has been widely confirmed [2, 8, 9]. Studies among men in a range of countries have also found low health literacy to be associated chronic disease morbidity [10–12].

Health literacy may be understood in general terms as being concerned with the capacities of people to address health issues (to ‘do health’) in a complex society [13]. However, its rapid growth and evolution has led to multiple interpretations of the concept, to the point that some view it as a source of confusion and debate [14, 15]. As a result, increasing attempts have been made to develop new, expanded and integrated models of health literacy [15–17].

Population studies in Australia [18], the United Kingdom [19] and the United States [20] have found men to have lower levels of health literacy than women, although other studies have not found a significant association [21] or have reported that gender differences in health literacy are moderated by age [22]. The lack of attention to potential confounders (e.g., income) is seen by some authors to be illustrative of the weaknesses of understanding health literacy generally and its relationship to sex (and gender) specifically [23]. A substantial body of literature describes the influence of gender upon health, which in the case of men may be shown in the way that health information is obtained, interpreted and applied [23, 24]. Manifestations of gender norms with repercussions for men’s health beliefs and actions include a social expectation of independence and control, embarrassment at showing vulnerability, and lack of communication with health professionals [25, 26]. However, while governments have adopted male-specific health policies and strategies, there has been little attention to health literacy and how this might be improved in order to reduce the burden of chronic disease in men [23].

Understanding the factors associated with men’s health literacy is necessary for tackling the preventable chronic diseases experienced by men. Indeed, it is recognised that gender appropriate strategies to promote health literacy are needed [7, 24]. This systematic review aims to address the knowledge gap that exists in the identification and understanding of the correlates of men’s health literacy and its components in relation to major lifestyle-related chronic diseases.

## Methods

A systematic review was conducted following the guidelines of the Cochrane Collaboration [27].

### Definition of health literacy

For the purposes of developing the search strategy, the integrated definition of health literacy derived by Sørensen *et al.* ([16], p.3) was used, which is: “people’s knowledge, motivation and competences to access, understand, appraise, and apply health information in order to make judgments and take decisions in everyday life concerning healthcare, disease prevention and health promotion to maintain or improve quality of life during the life course.”. From their systematic review of definitions and models of health literacy Sørensen *et al.* [16] identified a number of components of health literacy that were clustered under the heading of ‘competence, skills, abilities’. The definition of health literacy generated by Berkman *et al.* [17] placed a similar emphasis on abilities, or ‘know-how’, that can be put to use to communicate about health issues and make informed health decisions. Based on these definitions the terms ‘knowledge’, ‘competence’, ‘cognitive skill’, ‘social skill’ and ‘personal skill’ were selected, together with the U.S. National Library of Medicine’s Medical Subject Heading (MeSH) ‘Health Literacy’ and the related heading ‘Health Knowledge, Attitudes, Practice’ (or equivalents) for the exposure/intervention components of the search.

### Chronic diseases and related risk factors

The focus of the review was on the two highest causes of lifestyle-related chronic disease burden in men that share common risk factors, namely IHD and T2DM [1]. Currently IHD causes more than double the burden of disease among males compared with T2DM [8], but T2DM is projected to become the leading cause of disease burden in future decades [28]. IHD and T2DM were mapped to the biomedical (e.g., obesity, hypertension) and behavioural (e.g., smoking, physical inactivity, unhealthy eating) determinants of those conditions [2], and these factors were included as relevant topics in the database searches.

### Search strategy

The database searches for the review were based on the modified Population-Intervention-Comparator-Outcome-Study design (PICOS) framework as shown in Table 1.

**Table 1:** Modified PICOS framework

Population	Risk (or Outcome)	Exposure (Intervention)
Men, or	Health Literacy, or	Smoking, or
Male, or Masculine/Masculinity	Health Knowledge, Attitudes, Practice, or Cognitive Skill (and health), or Social Skill (and health), or	Physical inactivity, or Dietary imbalance/low fruit and vegetable intake, or
	Personal Skill (and health), or Knowledge (and health), or	Overweight and obesity, or Hypertension, or
	Competence (and health)	High blood fats, or Ischaemic heart disease, or
		Type 2 diabetes

Five databases were selected for the review and searched in August 2013: CINAHL, Medline, Embase, PsycINFO and the Cochrane Library. Each key term was searched by both heading and free-text terms. Additional studies were identified through reference and citation tracking.

### Inclusion and exclusion criteria

Included studies were those in which health literacy and its components (i.e., knowledge, attitudes, competence, skills, self-efficacy) were treated as dependent variables.

Eligible studies were those of observational or experimental design that were undertaken in a developed country [29], consistent with other reviews of health literacy research [16, 30].

Studies were excluded if they were of a paediatric population, if the article was focussed on women, or was not published in English. In addition, studies targeting populations of persons practising in a health occupation were also excluded. Studies were restricted to those published from 2003 onwards (ten years prior to the search date).

### Quality and strength of evidence assessment

Descriptive information about each study was extracted by one of the researchers (JD) and tabulated, including author and year, study design, sample and location, components of health literacy measured, variables included in multivariable analysis and significant correlates identified. The methodological quality of the included studies was assessed using criteria relevant to observational studies identified in the *Cochrane Handbook for Systematic Reviews of Interventions* [27], namely: risk of selection bias; adjustment for confounders; data collection methods (use of reliable or valid instruments); withdrawals and dropouts, and; analysis methods appropriate to the properties of the data. Each study was rated as strong, moderate or weak based on the assessment of its quality in each of these domains. The overall strength of the evidence for each correlate identified from all of the studies was rated as high, moderate, low or very low using the Grades of Recommendation, Assessment, Development and Evaluation (GRADE) [27]. The quality of evidence derived from sound observational studies is generally rated as low using the GRADE criteria, but may be upgraded to moderate or higher if there is a large magnitude of association, a consistency of association across studies, high precision of estimates, and no obvious biases that may explain these [27, 31]. Because of the heterogeneity of the studies in regards to measures of health literacy and its components, their correlates and the adjustment for potential confounders, it was not possible to quantitatively summarise the evidence using a meta-analysis.

## Results

**Fig. 1:**
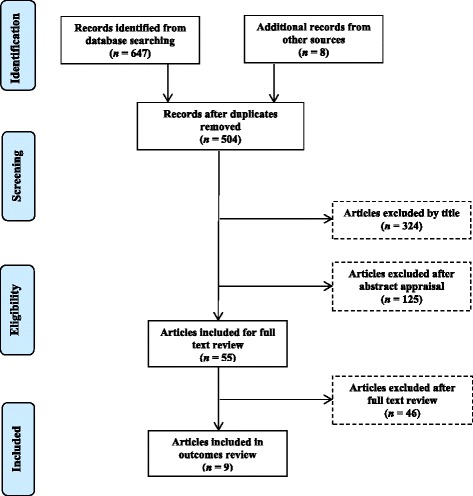
Flow diagram for study identification

The search strategy yielded 647 citations, and an additional eight citations were located through reference checking. The number of articles excluded at each stage is shown at Fig. 1. Exclusions at the full text review stage were largely due to data tables not reporting male-specific results (83.6 %). Nine articles were included in the final review.

### Characteristics of included studies

The characteristics of the included studies are summarised in Table 2. All nine of the included studies were observational and cross-sectional in design. Almost half (four of nine) of the studies were conducted in the United States. Three of the studies were conducted in north Asia (Japan, Taiwan and South Korea). One study was undertaken in Europe (Portugal) and one in Guadeloupe and Martinique (French West Indies).

**Table 2:** Characteristics of included studies

Study, Year (Reference)	Design	Study sample participants, location and size	Component of health literacy	Outcome measure	Variables in multivariate analysis	Significant correlates
Aihara and Minai 2011 [32]	Cross-sectional	Japanese men ≥ 75 years from Odawara City, Kanagawa Prefecture (*n* = 347)	Health Literacy (Nutrition)	Adequate nutrition literacy *vs.* inadequate nutrition literacy	Age, education, economic status, cognitive difficulty, sensory impairment, social network, information resources, self-rated health, BMI	Adjusted odds ratio (95 % CI):
						Informational support, 5.59 (1.28–24.49);
						Friends as source of information, 2.16 (1.11–4.20);
						BMI ≥ 25 kg/m^2^, 2.17 (1.20–3.91)
Kan and Tsai 2004 [39]	Cross-sectional	Taiwanese men from two townships (*n* = 1726)	Knowledge (Knowledge of health risks of obesity)	Probability of risk knowledge	Age, marital status, health, education, income, religion, vegetarian, work, housework, newspaper use, TV news, meets friends, community participation	Ordered probit coefficient (*t*-test):
						Education, IHD 0.06 (6.67),
						T2DM 0.05 (5.44);
						Income, IHD 0.01 (2.62);
						Newspaper reader, IHD 0.14 (2.15), T2DM 0.22 (3.46);
						Participates in community, IHD 0.14 (2.18), T2DM 0.15 (2.38)
Kelly-Irving *et al.* 2010 [36]^a^	Cross-sectional	French West Indian men (*n* = 465)	Knowledge (Knowledge of risk factors for and prevention of stroke, IHD)	Correct *vs.* incorrect identification of preventability and >1 risk factor for stroke or IHD	Not specified	Percent (adjusted p-value) Education, IHD knowledge, 64 % < = 6 yrs, 78 % 7-11 yrs, 80% > = 12 yrs (p<0.001)
Lutfiyya *et al.* 2008 [33]	Cross-sectional	U.S. men from 25 states/territories (*n* = 19,163)	Knowledge (Knowledge of heart attack and stroke symptoms)	Low knowledge *vs.* high knowledge	Age, education, health insurance, income, deferred medical care, primary care provider	Adjusted odds ratio (95 % CI):
						Age 18-34 years (*vs.* ≥55 years), 0.42 (0.42-0.42);
						Age 35-54 years (*vs.* ≥55 years), 0.24 (0.24-0.24);
						Education < high school, 2.42 (2.40-2.43);
						No primary care provider, 1.16 (1.15-1.16);
						Annual household income ≥ $35 k, 1.21 (1.21-1.22);
						Care deferred because of cost, 1.24 (1.23-1.24);
						No health insurance, 1.92 (1.91-1.93)
Lutfiyya *et al.* 2010 [34]	Cross-sectional	U.S. Hispanic men from 23 states/territories (*n* = 2023)	Knowledge (Knowledge of heart attack and stroke symptoms)	Low knowledge *vs.* high knowledge	Age, education, health insurance, income, deferred medical care, primary care provider	Adjusted odds ratio (95 % CI):
						Age 18-34 years (*vs.* ≥55 years), 0.26 (0.25- 0.26);
						Age 35-54 years (*vs.* ≥55 years), 0.38 (0.37- 0.38);
						Education < high school, 16.27 (15.74-16.82);
						No primary care provider, 2.05 (2.02-2.09);
						Annual household income ≥ $35 k, 0.96 (0.95-0.97);
						Care not deferred because of cost, 2.10 (2.06-2.14);
						No health insurance, 1.54 (1.52-1.57)
Murata *et al.* 2003 [35]^b^	Cross-sectional	U.S. Type 2 diabetic veterans from 3 VA clinics in 2 states (*n* = 180, 94 % male)	Knowledge (Diabetes knowledge)	Questionnaire raw score converted to per cent correctly answered	Age, years of schooling, treatment duration, MMSE score, depression score, sex	Linear regression coefficient (p-value):
						Age, −0.47 (<0.001);
						Years of schooling, 1.03 (0.003);
						Duration of treatment, 0.25 (0.03);
						MMSE score, 1.62 (0.001)
Periera *et al.* 2009 [37]	Cross-sectional	Portuguese hypertensive men from the city of Porto (*n* = 889)	Knowledge (Hypertension awareness)	Aware *vs.* unaware	Age, BMI, alcohol intake, triglycerides, diabetic, marital status, health care setting	Adjusted odds ratio (95 % CI):
						Age 16-60 year (*vs.* ≤ 15 years), 3.43 (1.68-7.00);
						Age ≥61 year (*vs.* ≤15 years), 3.69 (1.89-7.21);
						BMI 25–29 kg/m^2^ (*vs.* <25 kg/m^2^), 2.18 (1.35-3.52);
						BMI ≥ 30 kg/m^2^ (*vs.* <25 kg/m^2^), 2.86 (1.59-5.16);
						Not married, 0.45 (0.25-0.81)
Sohn *et al.* 2007 [40]	Cross-sectional	South Korean men hospitalised for CVD (*n* = 97)	Personal skill (Confidence in quitting smoking)	High confidence *vs.* low confidence	Age, education, marital status, alcohol dependence, age commenced smoking	Adjusted odds ratio (95 % CI):
						Married, 5.54 (1.33-23.08);
						CAGE score ≥2, 3.25 (1.20-8.80);
						Age commenced smoking ≤20 year, 2.96 (1.14-7.68)
Wyatt *et al.* 2008 [38]	Cross-sectional	U.S. hypertensive African American men from Jackson, Mississippi (*n* = 927)	Knowledge (Hypertension awareness)	Aware *vs.* unaware	Age, weight, smoker, T2DM, CVD, high cholesterol, health insurance,accesses preventive care	Adjusted odds ratio (95 % CI):
						BMI ≥30 kg/m^2^ (*vs.* < 25 kg/m^2^), 3.82 (1.79-8.11);
						T2D present, 2.82 (1.10-7.20);
						Preventative care, 4.32 (2.55-7.34);
						Current smoker, 0.29 (0.15-0.54);
						Age, 1.05 (1.02-1.07)

*BMI* Body Mass Index; *CVD* Cardiovascular disease; *IHD* Ischaemic Heart Disease; *MMSE* Mini-Mental State Examination; *VA* Veterans’ Affairs

^a^multivariate analysis not specified

^b^this study was treated as a male-specific study given the proportion of male subjects and the non-significance of the sex coefficient in multivariate analysis

Despite the broad search strategy, only one study examined health literacy (nutrition literacy) [32]. The majority of included studies focused on only one component of health literacy, namely knowledge (including awareness) (*n* = 7) and personal skills (confidence) (*n* = 1).

Three studies examined knowledge of disease, which was heart and stroke symptoms [33, 34] and T2DM [35]. The remaining six studies concerned risk factors for IHD and T2DM, including hypertension, nutrition, obesity and smoking [32, 36–40].

Seven studies measured differences in components of health literacy dichotomously and the remaining two used continuous measures, one as a percentage-correctly-answered measure [35] and the other a probit-based probability-of-awareness measure [39]. All but one of the studies that presented dichotomous results used multivariate logistic regression techniques. Kelly-Irving *et al.* [36] presented their results for knowledge of heart disease or stroke prevention by way of bivariate analysis only (using the *χ*^2^ test). The study by Kan and Tsai [39] used ordered probit model regression to calculate coefficients of awareness of the health risks of obesity.

Table 3 shows that seven of the studies were rated as moderate in quality, and the reminder as weak. Only one study, that included African Americans recruited in the Jackson Heart Study [38], was rated as strong in relation to the risk of selection bias. There was considerable variability in regard to control of potential confounders, with five rated as strong on this criteria [32–34, 38, 39]. All but one study controlled for age and only two studies did not control for education. The majority (five studies) did not control for income or economic status. The studies reported by Murata *et al.* [35] and Sohn *et al.* [40] used validated measures of health knowledge and personal skill, respectively. Four other studies used instruments that were adapted from previous, large-scale studies or surveys: CVDFACTS [39], MONICA [36] and the Behavioral Risk Factor Surveillance Survey (US Centers for Disease Control and Prevention) [33, 34].

**Table 3:** Ratings of methodological quality of included studies

Study	Risk of selection bias	Confounders	Data collection methods	Withdrawals and dropouts	Analysis	Quality rating
Aihara and Minai [32]	Mod	Strong	Weak	Mod	Mod	Mod
Kan and Tsai [39]	Mod	Strong	Weak	Mod	Mod	Mod
Kelly-Irving *et al.* [36]	Weak	Weak	Mod	Mod	Weak	Weak
Lutfiyya *et al.* [33]	Mod	Strong	Mod	Mod	Mod	Mod
Lutfiyya *et al.* [34]	Mod	Strong	Mod	Mod	Mod	Mod
Murata *et al.* [35]	Mod	Weak	Strong	Weak	Mod	Mod
Periera *et al.* [37]	Mod	Weak	Weak	Strong	Mod	Mod
Sohn *et al.* [40]	Weak	Weak	Strong	Weak	Mod	Weak
Wyatt *et al.* [38]	Strong	Strong	Mod	Mod	Mod	Mod

### Correlates of health literacy and its components

The factors found to have significant associations with health literacy or its components in two or more of the reviewed studies were education, age, health insurance status, income, marital status, overweight and obesity, and access to primary health care services. In addition, a diverse range of correlates were identified in single studies, including various measures of social connectedness, risk behaviours and information access.

### Education

Six of the nine studies found an association between educational attainment and components of health literacy (nutrition literacy, knowledge, personal skill) (Table 4). The direction of association was positive in all cases. Three of the studies did not control adequately for potential confounders [35, 36, 40]. The remaining three studies demonstrated large magnitudes of effects, narrow confidence intervals and strong control for plausible confounders [33, 34, 39]. The evidence for education as a correlate of components of men’s health literacy was rated as moderate.

**Table 4:** Strength of evidence concerning correlates of components of men’s health literacy

Correlate	Component	Study design	Number of studies (Adjusted:Unadjusted for confounders)	Results^a^		Evidence rating
				Adjusted	Unadjusted	
Education	Nutrition literacy; knowledge; personal skill	Cross-sectional	7 (4:3)	3	3	Moderate
Age	Knowledge	Cross-sectional	8 (6:2)	4	1	Low
Health insurance	Knowledge	Cross-sectional	2 (2:0)	2		Low
Income	Knowledge	Cross-sectional	3 (3:0)	2		Low
Marital status	Knowledge; personal skill	Cross-sectional	3 (3:0)	2		Low
Overweight or obese	Nutrition literacy; knowledge	Cross-sectional	3 (2:1)	2		Low
Primary care service access	Knowledge	Cross-sectional	2 (2:0)	2		Low
Accesses preventive care	Knowledge	Cross-sectional	1 (0:1)		1	Low
Community connected	Knowledge	Cross-sectional	1 (1:0)	1		Low
Friends source of nutrition information	Nutrition literacy	Cross-sectional	1 (1:0)	1		Low
Information from social supports	Nutrition literacy	Cross-sectional	1 (1:0)	1		Low
Regular newspaper reader	Knowledge	Cross-sectional	1 (1:0)	1		Low
Treatment duration	Knowledge	Cross-sectional	1 (1:0)	1		Low
Care deferred because of cost	Knowledge	Cross-sectional	2 (2:0)	2		Very low
Cognitive ability	Knowledge	Cross-sectional	2 (1:1)	1	1	Very low
Alcohol consumption	Personal skill	Cross-sectional	2 (2:0)	1		Very low
Age commenced smoking	Personal skill	Cross-sectional	1 (1:0)	1		Very low
Smoking status	Knowledge	Cross-sectional	1 (0:1)		1	Very low
T2 diabetic	Knowledge	Cross-sectional	2 (1:1)		1	Very low
Visually impaired	Nutrition literacy	Cross-sectional	1 (0:1)		1	Very low

^a^Number of studies that adjusted or did not adjust for confounders to find a significant association between the correlate and the component of health literacy

### Age

Five studies found an association between age and health knowledge. The direction of association in one study was negative, i.e., increasing age was associated with decreased health knowledge [33]. The stratified data in one study suggested a quadratic relationship between age and knowledge whereby knowledge improved from early adulthood to middle age, then declined after 55 years [34]. Two studies conducted amongst participants with hypertension indicated a positive association between age and health knowledge. However, lack of control for length of treatment in both studies increased the risk that those associations were confounded by duration of treatment [37, 38]. The study by Murata *et al.* [35], which was conducted in a population with T2DM, controlled for length of treatment and found a negative association between age and knowledge. Given these inconsistencies in the direction of association, the evidence for age as a correlate of men’s health knowledge was rated as low.

### Health insurance

Two studies found an association between lack of health insurance and knowledge, the first of which had an odds ratio close to 2.0 and a narrow 95 % confidence interval [33, 34]. Overall, the evidence for this correlate was rated as low.

### Income

The two studies with the largest sample sizes showed a negative association between income and health knowledge [33, 34]. However, a sub-group analysis by ethnicity in one of those studies [34] showed an odds ratio just below one (i.e., a very weak negative association) for one ethnic group and an odds ratio of almost two (i.e., a very strong positive association) for the other [34], with no explanation for this difference. Under the GRADE approach, an unexplained inconsistency such as this decreases the quality level of a body of evidence [27]. On balance, the evidence for income as a correlate of men’s health knowledge was rated as low.

### Marital status

Two of three studies [37, 40] that included marital status in their analysis found a positive association between this variable and different components of health literacy, namely knowledge [37] and personal skill [40]. The evidence for marital status as a correlate of components of men’s health literacy was rated as low.

### Overweight or obesity

One of the three studies reporting an association between Body Mass Index (BMI) and components of health literacy showed a positive association between obesity and health knowledge, but no association between overweight and knowledge [38]. In contrast, another study showed positive associations between knowledge and both overweight and obesity [37]. The third study, which measured the relevant association for BMI > 25 kg/m^2^ (i.e., overweight or obesity) showed a positive association between overweight or obesity and nutrition literacy [32]. The evidence for the association of overweight or obesity as a correlate of components of men’s health literacy was rated as low.

### Primary care service access

The two studies by Lutfiyya *et al.* [33, 34] found a positive association between men not having appointed a primary care provider and low heart attack and stroke symptom knowledge, i.e., low access was associated with low knowledge. The evidence for this association was much weaker in the first [33] compared to the second [34] study. Overall, the evidence for primary care service access as a correlate of knowledge was rated as low.

### Other factors associated with components of health literacy

Each of the following factors was identified in a single study as being associated with components of health literacy and the quality of that evidence was assessed as low: level of community-connectedness (knowledge) [39]; friends as the source of nutrition information (nutrition literacy) [32]; social supports as a source of information (nutrition literacy) [32]; regular newspaper reading (knowledge) [39], and; duration of treatment (knowledge) [35].

The following factors were identified as being associated with components of health literacy, however, the quality of the relevant body of evidence was assessed as being very low: care deferred because of cost (knowledge) [33, 34]; cognitive ability (knowledge) [35]; alcohol intake (personal skill) [40]; age commenced smoking (personal skill) [40]; current smoking status (knowledge) [38]; T2DM (knowledge) [38], and; visual impairment (nutrition literacy) [32]. The body of evidence for this group of correlates was downgraded to very low on the basis of weak or inconsistent associations; for example, the direction of association between care deferred because of cost and men’s health literacy was inconsistent between the two relevant studies [33, 34].

## Discussion

This systematic review identified 20 different correlates of components of men’s health literacy related to IHD and T2DM. The review also highlighted a significant shortage of studies into men’s health literacy or its components; only 9 of 55 candidate studies focused exclusively on men or stratified their results by sex. There has been little attention to health literacy as an enabler of preventive action to reduce disease burden in men. This may, to some extent, be the result of the lack of empirical evidence concerning men’s health literacy [23, 24]. A stronger understanding of this important health determinant will help to ensure that men’s health promotion strategies are appropriately targeted, with recognition of the health experiences of men reflected in the issues addressed, the language used, and the settings in which these are delivered [41].

While there is not yet consensus around a conceptual framework that defines the elements of health literacy and their relationships with downstream health outcomes [42, 43], the literature identifies a range of factors associated with personal health literacy. Sørenson *et al.* [16] have identified general literacy, prior experiences with illness and the healthcare system, socioeconomic status, age, race, gender, verbal ability and reasoning, numeracy, physical abilities, social skills, occupation, employment status, income, levels of social support, language and cultural background as ‘antecedents’, ‘factors which impact’, or ‘predictors’ of health literacy. Paasche-Orlow and Wolf [44] depict similar factors, along with verbal ability and reasoning as being ‘strongly associated’ with health literacy. Peerson and Saunders [23] also note that both social and individual factors are ‘influential’, with health literacy skills ‘affected by’ factors such as education, culture, language, family and social relationships, as well as by the media, the market place and the provision of health information by agencies.

The variability in definitions and measures used in health literacy research has limited the ability to compare studies and advance the field to develop effective interventions targeting population groups with low health literacy [45, 46]. The majority of studies that met the inclusion criteria for this review focused on a single component of health literacy, namely knowledge. While it is difficult to make definitive conclusions relevant to men’s health literacy *per se*, the correlates identified in this study have assisted in identifying the evidence gaps about key components of men’s health literacy and have the potential to be applied to the development of a health literacy framework specifically targeting men.

Ten of the correlates identified in this study matched, or were closely analogous, to the determinants of health literacy identified in these previous reviews and commentaries [16, 23, 43], namely: education; age; income; marital status; treatment duration; ‘health insurance’ and ‘care deferred because of cost’ (as proxies for economic status), and; ‘community connected’, ‘friends as a source of nutrition information’ and ‘information from social supports’ (as components of family and social relationships). The remaining correlates identified were predominantly health behaviours, biomedical factors or indicators of health status, which are often situated as outcomes of health literacy within established health determinant frameworks [16, 43, 47]. For example, obesity and overweight was found to be associated with health knowledge and nutrition literacy, as it is most likely that these components of health literary are antecedent influences upon weight status.

Education was the only factor supported by a body of evidence that was graded as moderate or greater strength. All of the other identified variables were supported by evidence graded as either low or very low. The degree of heterogeneity in the correlates identified in this review is noteworthy: 20 correlates were identified, but only two (age and education) were supported by evidence from more than two studies. Furthermore, two studies did not control for educational attainment, and more than half of the studies did not control for income or economic status. The omission of these variables increased the risk of bias in the studies and further illustrates how the lack of a consensus on a conceptual framework hinders the development of a body of evidence about the potential correlates of health literacy.

Prior illness and contact with the healthcare system have been identified as possible correlates of health literacy [16], and this review also identified that access to primary health care services and preventive care may be correlates of men’s health knowledge. A disparity exists in access to primary health care services between men and women, particularly younger men [48], with a number of individual, social, systemic and environmental factors potentially impacting on help-seeking behaviours [49]. Given that the association between low health literacy and poorer use of health services is well recognised [46], further research is also warranted to explore the causal relationship between health literacy and access to primary health care services for men. Adopting a ‘gender lens’ may identify alternative health promotion strategies within the primary health care setting to support men to better understand and use health information [24].

While it has been observed that the number of articles concerning health literacy in the academic literature has grown markedly since the 1990s [43], in this review it was found that electronic retrieval of studies on this topic was difficult. It has been claimed as late as 2009 that some authors still did not use the term for relevant articles [30]. This was one reason this review adopted an expanded definition to include the components of health literacy. In spite of this, relevant and useful studies may have been missed because of this diversity in definitions and measures of health literacy. Given the rapid evolution of the concept of health literacy, the 10-year restriction imposed on the search strategy was deemed appropriate and reflects the practice of other systematic reviews [50]. However, this criterion may have excluded research that would have been informative. Likewise, the restriction to articles published in English is common [46], but may again have resulted in the exclusion of useful sources.

## Conclusion

Understanding the correlates of men’s health literacy and its components provides knowledge about those areas where policy and practice interventions may be directed to support men’s help-seeking behaviours and health outcomes [24]. The review identified comparatively few and relatively weak quality studies on men’s health literacy, focusing primarily only on knowledge components, despite a broad search strategy. Research on the correlates of health literacy appears to have been hampered by a lack of a consensus understanding of the nature and scope of the term. This lack of consensus has contributed to the absence of a standard measure (or suite of measures) and a conceptual framework concerning health literacy. To better understand the correlates of men’s health literacy and its components, further research is needed to develop the foundations of the health literacy field more broadly with consensus definitions, validated instruments and conceptual frameworks. With these building blocks in place, more focussed and well-designed studies into the correlates of men’s health literacy and its components can be undertaken, to inform evidence-based recommendations for men’s health policy and practice.
